# Evaluation of the clinical pharmacist services at a gynaecological oncology preadmission clinic

**DOI:** 10.1016/j.rcsop.2022.100213

**Published:** 2022-12-16

**Authors:** Natasha Triscari, Stephanie Wai Khuan Teoh, Marcus Femia

**Affiliations:** Pharmacy Department, Perth, Western Australia 6008, Australia

**Keywords:** Medication safety, Medication reconciliation, Preadmission clinic, women health, pharmacist

## Abstract

**Background:**

Pharmacists working in the multidisciplinary gynaecological oncology pre-admission clinic (PAC) are involved in the perioperative assessment of patients for a comprehensive medication history and information provision regarding withholding of medications before surgery.

**Objective:**

To evaluate the current services provided by pharmacists to multidisciplinary staff and patients attending the PAC.

**Methods:**

A staff and a patient feedback survey on the value and impact of PAC pharmacy services were distributed within the PAC. The impact of the PAC pharmacist was also assessed by analysing pharmacist interventions and key performance indicators documented.

**Results:**

Fifteen staff responses were recorded, 5 nursing staff, 2 midwives and 8 anaesthetists. Eighty-seven percent (*n* = 13) strongly agreed or agreed that pharmacists at PAC help reduce medication errors on admission. Staff strongly agreed 73% (*n* = 11) pharmacists obtain a more accurate medication history. Staff reported benefits in having a pharmacist at the clinic to discuss medication related questions with 87% (*n* = 13) strongly agreeing or agreeing with the statement. A staff overall satisfaction rating of 4.87 out of 5 was recorded. In the patient survey, respondents (*n* = 6) gave a 4.83 out of 5 rating in confidence in making changes to their medication and their overall satisfaction with the service provided. In reviewing data from January to June 2022, the number of patients seen by the pharmacist were 178 of 681 patients (26.1%) who attended the clinic. The most common medications involved in the pharmacist intervention include those that were advised to be withheld and those that required other changes to therapy prior to their procedure.

**Conclusion:**

The role of a PAC pharmacist can be greatly appreciated by the multidisciplinary team and patients. Pharmacist interventions and key performance indicators have demonstrated the important activities of clinical pharmacy services in the PAC in optimising patient care in medication management.

## 1 Introduction

To prepare a patient for upcoming surgeries and procedures, appointments are made for the Preadmission Clinic (PAC) in order to adequately assess the patient and to provide appropriate education to them on how to initiate these preparations.^1^ This includes consultations with nursing staff as to fasting or fluid restrictions prior to the procedure, consultations with anaesthetists as to the most appropriate anaesthetic options for the patient, or discussion with the pharmacist regarding their medications that may require changes before the procedure.^2^ When this role is executed effectively, the resulting outcome is increased cost-effectiveness by a reduction in length of stay and admission time, as well as a reduction in unnecessary investigations, tests or consultations that would ultimately delay procedures.^1^^,^^2^

Clinical pharmacists play a significant role in endorsing the optimal use of medicines for patients on admission, during their stay in hospital as an inpatient, and upon discharge.^3^ PAC pharmacists have shown to minimise medication errors that can occur on the day of hospital admission.^3^ Pharmacists at PAC engage in patient interviews to identify medications the patient uses regularly and when required, screen for and advise on medications to be withheld prior to surgery, and develop an accurate medication list that can be utilised when the patient is admitted.^4^ The value of this service is an increase in productivity and efficiency, reduced delays in procedures, reduced costs due to procedure cancellations or medication misuse and an accurate medication history that can be used throughout their hospital journey.^5^^,^^6^

Numerous studies have demonstrated the significance and value of clinical pharmacist services at PAC.^6^, ^7^, ^8^, ^9^, ^10^ For instance, a study in the United Kingdom compared the interventions made by the PAC pharmacist with those made by the clinical pharmacists and reported that the interventions made by the PAC pharmacist has higher clinical significance, and lower medication prescribing errors including medication omissions.^10^ In Australia, a study showed that patient-completed medication histories in a PAC were inaccurate in 80% of cases which reflected the vital role of PAC pharmacist in obtaining an accurate medication history prior to admission.^7^ However, the value of the PAC pharmacist in a gynaecology setting has not been studied extensively. Furthermore, little is known on the multidisciplinary team's and patient's perspectives and satisfaction levels of the PAC pharmacy service provided by pharmacists.

The 300-bed (including 100 neonatal cots) study hospital is the only tertiary maternity and gynaecological hospital in Western Australia. More than 6000 births take place annually and it is the only major referral centre in the state for high-risk pregnancies. The hospital also provides services to approximately 5000 women with gynaecological conditions each year, including malignant and non-malignant urological problems, sexually transmitted diseases and reproductive disorders. Pharmacists at the study hospital have been involved in the assessment of high-risk patients at the gynaecological oncology surgical PAC since 2007.

## 2 Aim

The aim of this study was to obtain an understanding of the value and impact of the PAC pharmacist as part of the multidisciplinary team in a gynaecological oncology PAC. In obtaining the overall satisfaction and activities of the service, the pharmacists involved can continue to provide aspects of this service that are desired and valued, as well as potentially modifying aspects that may be identified as areas for improvement. Objectives were to: (1) assess satisfaction and added value by the PAC pharmacists from multidisciplinary team and patients' perspectives with a survey completed by the staff and patients respectively, and (2) to evaluate the key performance indicators documented by the PAC pharmacists which include the number of patients seen, the number of the patients deemed high-risk requiring interview and the common medications involved in clinical interventions made by the pharmacist.

## 3 Methods

### 3.1 Staff and patient surveys

A literature search was performed in the PubMed® database using key words such as pharmacist impact, surgical preadmission clinics, perioperative medication management and pre-operative assessment. These key words identify the pharmacist services provided at the preadmission clinic and they are also descriptors of benefits obtained from these interventions. Two separate surveys were composed targeting healthcare professionals and patients respectively to capture a more targeted feedback. An online survey for the multidisciplinary team through Microsoft Forms® was composed and displayed at the clinic, asking questions about services currently provided by pharmacists (Appendix 1). The survey included statements seeking level of agreeance, an overall rating of satisfaction, as well as an area for health professionals to write recommendations to improve the service. The link to the online survey was shared with the anaesthetists and nursing staff working in PAC from May to July 2022.

A paper survey was created and offered to patients who had interviews with the pharmacist, asking patients to rate their agreeance with statements on a scale of 1 to 5 with 1 being the least and 5 being the most agreeable (Appendix 2). Patients were also provided space to leave suggestions for improvements about the service and/or to provide input about the services received from the pharmacist. The paper survey was offered to the patients in PAC after they were seen by the PAC pharmacist from May to July 2022.

### 3.2 Evaluating KPIs and interventions

In order to assess the KPIs and common interventions made by the clinical pharmacists, the data from the online recording system in REDCap® were reviewed and collated to represent the top ten medications commonly seen at PAC. Additionally, the data that were collected and recorded in Microsoft Excel® pertaining to the number of patients seen and the number of patients attending the clinic were reviewed to determine the overall impact of the service.

### 3.3 Ethical statement

Human Research Ethics approval was obtained from the Women and Newborn Health Service Quality Improvement Committee on the 5th May 2022 (Approval number: GEKO 46580) at King Edward Memorial Hospital.

## 4 Results

### 4.1 Multidisciplinary team survey

**Fig. 1:**
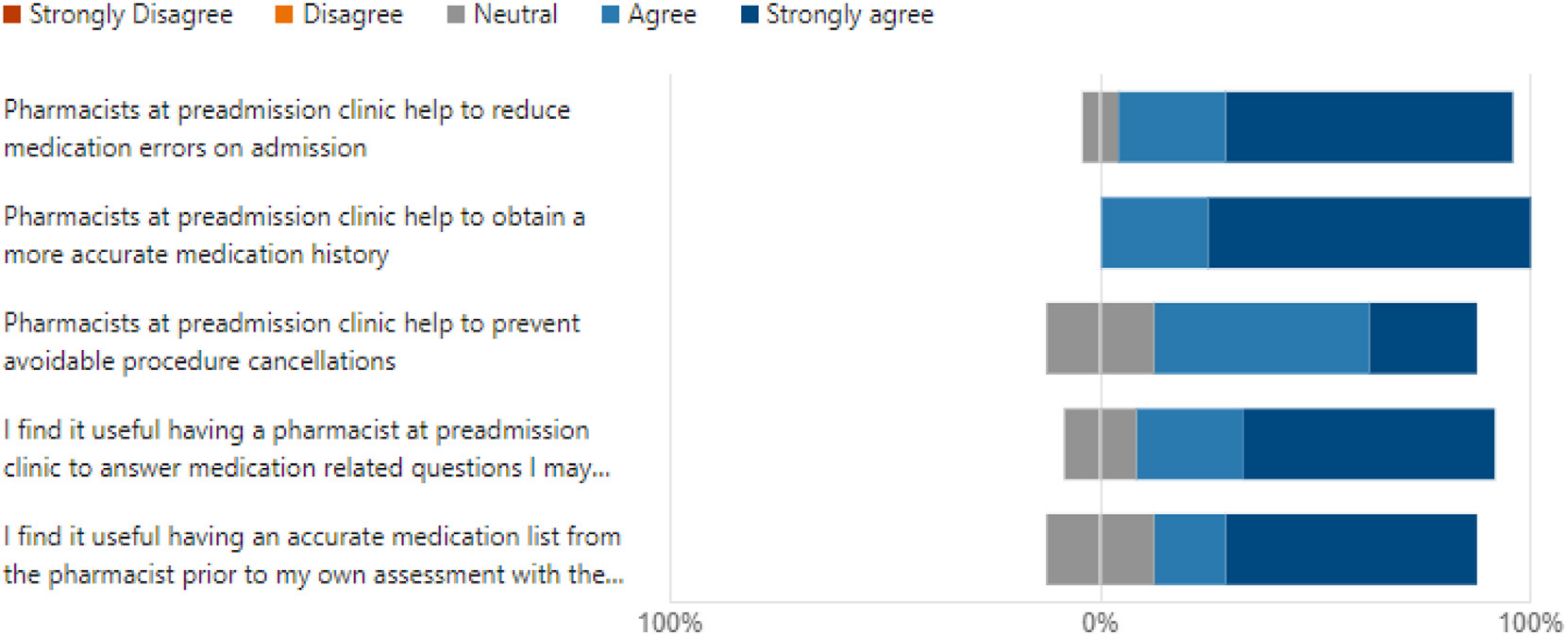
Multidisciplinary team agreement with statements on pharmacy services at the Pre-Admission Clinic.

**Fig. 2:**
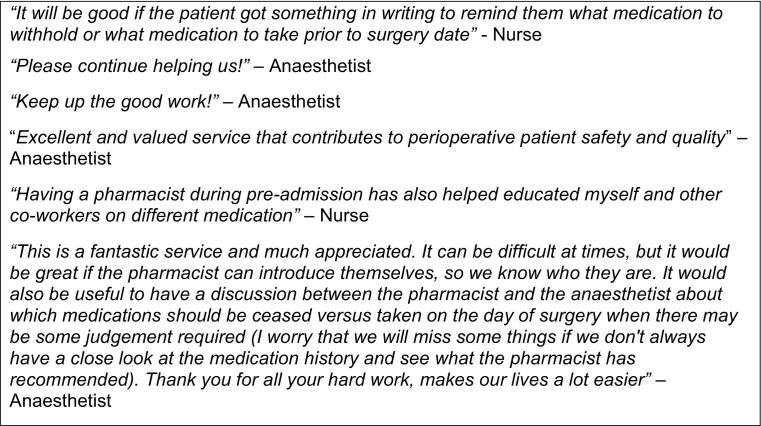
Staff feedback provided about the pharmacist services at pre-admission clinic and how it could be improved.

Over the time period of the evaluation, 15 responses were recorded by staff at PAC including 8 anaesthetists and 7 nursing or midwifery staff. Of the responses, an average rating of 4.87 out of 5 was reported for overall satisfaction with the service currently provided by pharmacists within the clinic (Fig. 1). Staff were asked to rate their level of agreeance with several statements, resulting in varying answers from ‘neutral’ to ‘strongly agree’. In response to the statement “pharmacists help to reduce medication errors on admission”, 27% of responses agreed, 60% of responses strongly agreed and 13% of responders were neutral to the statement. In response to “pharmacists help to obtain a more accurate medication history”, 73% of responders strongly agreed and 27% agreed. Additionally, staff were asked to determine whether they found it useful to have an accurate medication list from the pharmacist prior to their interview in which 20% reported being neutral, 27% agreeing and 53% strongly agreeing. Finally, when given the opportunity to provide written comments on the service currently provided, 50% of responders left comments with their suggestions to improve the service (Fig. 2).

### 4.2 Patient response survey

During the evaluation, 1 electronic survey and 5 paper-based surveys were completed by patients who were interviewed by a pharmacist as part of their PAC appointment. Patients were asked to identify how well they understood the pharmacist's role at PAC both before and after interview, with an increase from 4 to 4.67 out of 5. They were asked to identify their level of understanding of medications pre- and post-interview; again, an increase from 4.17 to 4.50 was reported. The survey asked patients to report how confident they felt in making changes to their medications as directed by the pharmacist and their overall satisfaction with the service provided to which responses were 4.83 out of 5 rating in both categories. Finally, the patients were asked to leave any comments relating to what aspect of seeing the pharmacist they valued the most, with 66.6**%** of responders leaving comments. None of the responders left comments on recommendations to improve the service.

### 4.3 Evaluating KPIs and pharmacist interventions

In reviewing data from January to June 2022, the number of patients seen by the pharmacist were 178 of 681 patients (26.1%) who attended the clinic. Of these 178 patients, 119 (66.9%) were deemed to be high-risk. The remaining 59 patients were made up of low risk overnight stay and day stay patients.

**Fig. 3:**
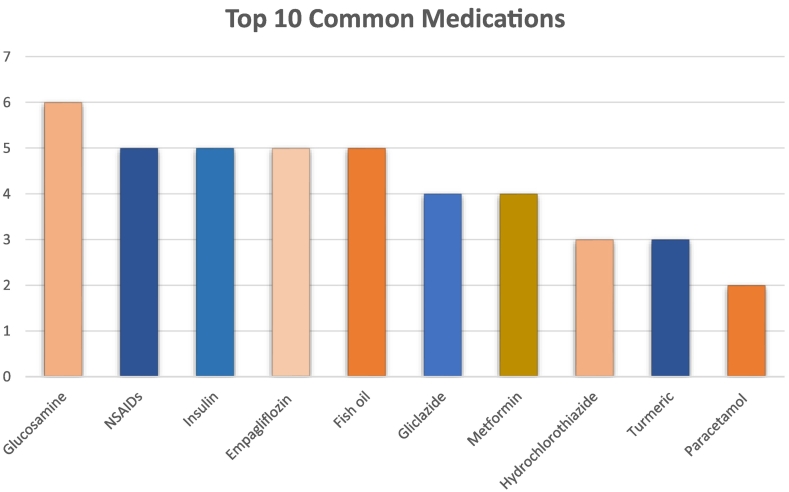
Common medications involved in the pharmacist interventions at Pre-Admission Clinic.

When reviewing the clinical interventions made by the pharmacist from a 3-month time period, the data were analysed to determine which medications were most involved in pharmacist interventions. These medications included those that were advised to be withheld, those that required other changes to therapy prior to their procedure, or medications that the patient had questions for the pharmacist about. The most common medication that was identified during the clinical interventions was glucosamine, followed closely by non-steroidal anti-inflammatories, insulins, empagliflozin and fish oil. Fig. 3 summarises the number of clinical interventions recorded during this time period relating to medications, with 6 interventions involving glucosamine and 5 interventions each involving the other top 5 medications listed previously. Other medications that were commonly implicated included, but were not limited to, gliclazide, metformin, hydrochlorothiazide, turmeric and paracetamol.

## 5 Discussion

This is the first study evaluating multidisciplinary team's and patient's perspectives and satisfaction levels of the PAC pharmacy service provided by pharmacists. The responses to the multidisciplinary team survey identified a trend in that the anaesthetic and nursing/midwifery staff agreed that a pharmacist involvement at the clinic is of value and helpful from a multidisciplinary approach. One finding of significance relates to the statement, “pharmacists help to obtain a more accurate medication history”, with all responders agreeing with this statement to some extent and 73% strongly agreeing. Another finding of the survey was that some nursing and midwifery staff tended to select “neutral” in response to statements rather than agree or disagree while the anaesthetists indicated their agreeance with all statements. It is possible that the anaesthetists felt more benefit and were more inclined to encourage a pharmacist input at PAC in order to prevent these cancellations and enhance efficiency within the hospital. The results of the study could be used as a benchmark for future similar studies especially for PAC in gynaecology setting.

In addressing patients' perspectives of the service provided, there was also a common trend that they felt the interaction with the pharmacist overall improved their understanding of medications. This trend was identified through patients expressing confidence in applying the changes to their therapy, feeling as though their knowledge of their medications improved after the interview, and understanding the pharmacist role better following the interview. Regarding patients feeling confident in their abilities to make changes to their therapy prior to their scheduled procedures, it also highlights how interactions such as these can help prevent patient errors and medication issues that may otherwise delay their procedures or compromise their safety. The results also indicated that the role of a preoperative pharmacist is greatly appreciated by patients.

By assessing the KPIs documented, the impact the pharmacist is having on the overall health of patients and efficiency of the hospital systems is apparent. A high proportion (93%) of high-risk patients were seen by the pharmacists, to have detailed medication histories taken within the PAC allowing for a faster reconciliation between medication history and charted medications when the patient is admitted. This creates a more time efficient process for clinical pharmacists on admission.^8^ This is particularly important for high risk patients that are at a higher risk of charting errors on admission.^8^ The part-time nature of the pharmacy service has limited the capacity of the PAC pharmacist meaning its full potential could not be evaluated and only high risk patients were screened and prioritised by the PAC pharmacist.

In reviewing the common drugs implicated in clinical interventions from PAC, it allows pharmacists, particularly working in gynaecology PAC to target their future practices and staff training towards these medications that are commonly used over time. In analysing the data, it became apparent that several complimentary medications such as glucosamine, fish oil and turmeric were becoming more common in patient's medication lists, likely due to increasing publications about their effectiveness or reported indications. Despite these medications carrying a theoretical risk of bleeding in contrast to anticoagulant or antiplatelet medications, the recommendations from current perioperative guidelines indicate the need for medications such as these to be withheld. This further enhances the importance of these being identified by the pharmacist and information passed on appropriately. In addition to this, there is a theme amongst the common medications with many of them being utilised for diabetes mellitus including insulins, empagliflozin, metformin and gliclazide. This trend of diabetic medications is not unexpected due to the varying fasting requirements for certain procedures, increasing diagnoses of diabetes mellitus in the community and additional risks associated with their use perioperatively such as with empagliflozin. Our pharmacist intervention study is unique as we evaluated the interventions made at the preadmission clinics. This is different to previous intervention reports which compared the pharmacist interventions required for admitted patients who have seen a pharmacist at PAC with admitted patients who have not seen a pharmacist at PAC.^3^^,^^6^^,^^8^^,^^10^

The data on pharmacists' interventions and KPI are dependent on the self-reporting of individual pharmacists which may have self-selection bias that may limited generalisability. Pharmacist interventions are often reported to be under-documented.^11^ Furthermore, the number of surveys received from patients is relatively small and does not represent all at PAC. During the evaluation, several PAC appointments were moved to telephone consults due to the COVID-19 pandemic. The paper-based surveys were only able to be distributed to those higher risk patients who attended the clinic in person. To overcome this, an electronic version was developed using Microsoft Forms® and distributed to those during telephone consults. Due to the patient cohort, some patients were unable to do the survey electronically with limited access to electronic devices. The data were collected at one site that specialises in women's health; the results are not generalisable beyond the patient population studied.

## 6 Conclusion

The role of a pre-admission clinic pharmacist can be greatly appreciated by the multidisciplinary team and patients. Patients' understanding of both their medicines and the pharmacist's role has been shown to improve after seeing the pharmacist at PAC. The pharmacist interventions reflected the positive contribution of the pharmacists in the management of patient medication use in the pre-and peri-operative period. The study is valuable in the continuing development and evaluation of PAC services in the study hospital and other hospitals providing gynaecology PAC pharmacist services.

## Funding

This research did not receive any specific grant from funding agencies in the public, commercial, or not-for-profit sectors.

## Ethical statement

Human Research Ethics approval was gained from the Women and Newborn Health Service Quality Improvement Committee on the 5th May 2022 (Approval number: GEKO 46580) at King Edward Memorial Hospital.

## CRediT authorship contribution statement

**Natasha Triscari:** Methodology, Data curation, Methodology, Visualization, Writing – original draft, Formal analysis. **Stephanie Wai Khuan Teoh:** Conceptualization, Methodology, Writing – review & editing. **Marcus Femia:** Writing – review & editing.

## Declaration of Competing Interest

The authors declare that they have no known competing financial interests or personal relationships that could have appeared to influence the work reported in this paper.

## Appendix A Supplementary data

Supplementary material 1 Image 1

Supplementary material 2 Image 2
