# Supplementary material for: Evaluation of the clinical pharmacist services at a gynaecological oncology preadmission clinic
Source: Explor Res Clin Soc Pharm. 2022 Dec 16;9:100213. doi: 10.1016/j.rcsop.2022.100213 (PMC9791024; doi:10.1016/j.rcsop.2022.100213)
Supplement: Supplementary file 1 — Supplementary material 1 [file mmc1.pdf]

PATIENT SURVEY

PRE-ADMISSION CLINIC PHARMACY

Please answer the following questions by placing a CIRCLE around the number that best describes your response (1 being the lowest and 5 being the highest). Participation in this survey is voluntary and all responses are kept anonymous.

After completing the survey, please return it to the BOX ON THE RECEPTIONIST'S DESK.

**Question 1:** How well did you understand the role of the pharmacist before attending the pre-admission clinic?				
1	2	3	4	5

**Question 2:** How well did you understand the role of the pharmacist after attending the pre-admission clinic?				
1	2	3	4	5

**Question 3:** How well did you understand your medications and how to use them, before being seen by the pharmacist?				
1	2	3	4	5

**Question 4:** How well did you understand your medications and how to use them, after being seen by the pharmacist?				
1	2	3	4	5

**Question 5:** How clearly was any advice communicated by the pharmacist?				
1	2	3	4	5

**Question 6:** How confident do you feel in making the changes to your medications recommended by the pharmacist, prior to your upcoming procedure?				
1	2	3	4	5

**Question 7:** Overall, how satisfied were you with the service you received from the pharmacist at the pre-admission clinic?				
1	2	3	4	5

Question 8: What aspect of being seen by the pharmacist did you value the most? (Please describe your response below)-

____
____
____
____
____
____

Question 9: Do you have any suggestions for the pharmacist on how to improve the health services they provide? (Please describe your response below)-

____
____
____
____
____
____

Thank you for completing the Pre-Admission Clinic Pharmacy Survey ☺
