# Supplementary material for: Evaluation of the clinical pharmacist services at a gynaecological oncology preadmission clinic
Source: Explor Res Clin Soc Pharm. 2022 Dec 16;9:100213. doi: 10.1016/j.rcsop.2022.100213 (PMC9791024; doi:10.1016/j.rcsop.2022.100213)
Supplement: Supplementary file 2 — Supplementary material 2 [file mmc2.pdf]

Preadmission Clinic Pharmacy Survey for PAC Staff

Pharmacists have been involved in the clinical service delivery at KEMH gynaecology and oncology preadmission clinics for a number of years. Our goals in providing this service are to obtain an accurate medication history for high risk patients, screen for and advise on the withholding of medication prior to surgery and reduce delays in scheduled procedures. We would like to know how other health professionals at preadmission clinic view the pharmacists role at the clinic and would like to hear your feedback/suggestions on ways we can continue to improve this service into the future. Thank you for your time in completing this survey, your responses will remain anonymous and are very much appreciated!
Kind regards,
KEMH Pharmacy Department

1. What is your role at KEMH?

- ○ Nurse
- ○ Midwife
- ○ Anaesthetist
- ○ Allied Health
- ○ Other

2. Which areas of preadmission clinic do you work in?

- ☐ Gynaecology
- ☐ Oncology

3. Please indicate your level of agreement with the following statements regarding the clinical pharmacist service at preadmission clinic.

	Strongly Disagree	Disagree	Neutral	Agree	Strongly agree
Pharmacists at preadmission clinic help to reduce medication errors on admission	○	○	○	○	○
Pharmacists at preadmission clinic help to obtain a more accurate medication history	○	○	○	○	○
Pharmacists at preadmission clinic help to prevent avoidable procedure cancellations	○	○	○	○	○
I find it useful having a pharmacist at preadmission clinic to answer medication related questions I may have	○	○	○	○	○
I find it useful having an accurate medication list from the pharmacist prior to my own assessment with the patient (where possible)	○	○	○	○	○

4. Please rate your overall satisfaction with the service provided by pharmacists at preadmission clinic.

☆ ☆ ☆ ☆ ☆

5. Do you have any further comments, feedback or suggestions on this service and how it could be improved?

This content is neither created nor endorsed by Microsoft. The data you submit will be sent to the form owner.

Microsoft Forms
